# *Biophysical Reviews’* Topical issue: The 7th Nanoengineering for Mechanobiology Symposium 2024 Camogli, Genoa, Italy

**DOI:** 10.1007/s12551-024-01193-7

**Published:** 2024-04-23

**Authors:** Costanza Giampietro, Aldo Ferrari, Massimo Vassalli

**Affiliations:** 1 EMPA, 8600 Dübendorf, Switzerland; 2 Hylomorph AG, Zurich, Switzerland; 3 James Watt School of Engineering, University of Glasgow, Glasgow, UK

## Abstract

This Commentary describes an open call for submissions to the upcoming Biophysical Reviews’ Issue Focus: The 7th Nanoengineering for Mechanobiology (Genova, Italy). The submission deadline is August 1st of 2024. Interested parties are requested to make contact with the Issue Focus editors prior to submission.

Every 2 years, mechanobiology enthusiasts from all over the world convene in Camogli (Genova, Italy) for the Nanoengineering for Mechanobiology (N4M) symposium. In 2024, the 7th N4M Congress was held between the 3rd and the 7th of March. Details of the conference can be found at the following website:

http://n4m.mechanobiology.eu/2024.

A few months following the meeting, the IUPAB journal *Biophysical Reviews* will collect articles for an Issue Focus on the topics presented at the 7th N4M symposium. The Issue Focus will be based principally on articles commissioned from the Congress speakers. However, contributions will also be considered from scientists who participated to the previous editions of the symposium, and those making poster presentations, after suitable consultation with the Special Issue Editors.

## Submission deadline

Review articles are requested to be submitted prior to August 1st in order to be considered for online publication in Issue 6 of that same year. When submitting your article, please make sure to choose the correct special issue assignment

## Special issue editors

Costanza Giampietro, Aldo Ferrari, Massimo Vassalli

## Email contact

Costanza Giampietro: costanza.giampietro@empa.ch

Massimo Vassalli: massimo.vassalli@glasgow.ac.uk

## Submission formats

Three general types of articles will be considered:

1. *Short letters*—300 to 600 words with one or two figures that provide a short description of a topical field in biophysics or an event of significance/relevance to the congress. These types of submissions are particularly encouraged from prominent members of the scientific community and relevant society members. It is requested that these letters be either of an educational, historical, or topical nature.
2. *Session commentaries* (written by congress session chairpersons)—300 to 600 words with one or two figures describing the theme of their session and the topics and backgrounds of the speakers appearing in their session.
3. *Review article*s—Two types of review article formats will be considered: a short review (1000–4000 words, three figures) or a long review (5000–10,000 words, approximately ten figures).

## About *Biophysical Reviews*

*Biophysical Reviews* was launched in 2009 and is the official journal of the ‘International Union for Pure and Applied Biophysics’. *Biophysical Reviews* publishes both short and long review formats from key figures in the field. The majority of contributions are invited although interested authors are encouraged to discuss with the Editor the possibility of contributing a review article. *Biophysical Reviews* covers the entire field of biophysics, generally defined as the science of describing and defining biological phenomenon using the concepts and techniques of physics. This includes but is not limited by such areas as bioinformatics, biophysical methods and instrumentation, medical biophysics, biosystems, cell biophysics and cellular organization, macromolecule dynamics, structure and interactions, single molecule biophysics, membrane biophysics, channels and transportation.

## Journal homepage

Http://www.springer.com/journal/12551.

## Online submission

Https://submission.springernature.com/new-submission/12551/3.

## Cost

*Biophysical Reviews* is a hybrid open-access journal and therefore operates on a two-track payment structure. The first track known as the ‘subscription option’ is completely free but requires that the article exist behind a paywall for approximately 6 months whilst the second track, known as ‘gold open access’, involves an open access charge of 2500 Euros. Both tracks give a highly desirable publishing outcome for the author. The choice of free subscription or paid open access is decided by the author following acceptance of their article. Please contact the Special Issue editors for further explanation of this point.

## References

[CR1] N4M http://n4m.mechanobiology.eu. Accessed 20 Jan 2024

